# Tissue-autonomous pharmacological direction: how target expression landscapes convert balanced compounds into tissue-selective agents

**DOI:** 10.3389/fphar.2026.1870571

**Published:** 2026-06-30

**Authors:** Huizhen Chen, Qiu Chen, Yanzhong Wang

**Affiliations:** 1 Chengdu University of Traditional Chinese Medicine, Chengdu, China; 2 King’s College London, London, United Kingdom; 3 Hospital of Chengdu University of Traditional Chinese Medicine, Chengdu, China

**Keywords:** direction reversal, multi-target drugs, polypharmacology, precision medicine, tissue direction index, tissue-autonomous pharmacological direction, tissue-specific drug effects, transcriptomic atlas

## Abstract

Multi-target drugs frequently exhibit tissue-dependent pharmacological directions: the same compound may promote a biological process in one organ while driving its functional opposite in another. Tamoxifen antagonizes estrogen receptors in breast tissue yet activates them in the endometrium; non-selective NSAIDs reduce inflammation at injury sites while damaging the gastric mucosa. Each phenomenon has been explained by drug-specific mechanisms, but no unifying principle predicts when or why such direction reversal occurs. Here we propose tissue-autonomous pharmacological direction (TAPD): when a compound simultaneously engages two functionally opposing target classes, its net pharmacological direction in any tissue is determined primarily by which class is more abundantly expressed locally. We formalize this principle through the Tissue Direction Index (TDI), a dimensionless metric (0–1) integrating binding affinity with tissue-specific expression data from transcriptomic atlases. As proof of concept, we show that bridging metabolites from traditional Chinese botanical drugs with uniform molecular-level coagulation bias undergo substantial hemostatic direction shifts between gastrointestinal and brain tissue (4.6-fold Hemostatic Index change, driven predominantly by expression differences), with two metabolites achieving complete direction reversal. We then discuss how TAPD complements existing models of tissue-selective drug effects, including SERMs, NSAIDs, kinase inhibitors, and immunomodulators, and outline translational applications in drug repurposing, predictive toxicology, and patient-specific pharmacological prediction.

## 1 Introduction

During my first year of clinical residency, I managed an elderly patient who presented with concurrent gastrointestinal hemorrhage and acute cerebral infarction. The therapeutic dilemma was absolute: hemostasis in the gut demanded procoagulant intervention, while the ischemic brain demanded the opposite. Despite our best efforts, the paradox proved irreconcilable, and we lost him. That experience left a lasting question: could a single therapeutic agent ever achieve hemostasis at one anatomical site while simultaneously promoting blood flow at another?

This clinical paradox is not an isolated anecdote. Concurrent GI bleeding and ischemic stroke affect a substantial proportion of elderly patients, with anticoagulant-associated GI hemorrhage occurring at rates of 2%–5% per year (Weil et al., 1995; Lansberg et al., 2012). More broadly, the scenario exemplifies a pervasive yet undertheorized phenomenon in pharmacology: a single compound producing functionally opposite effects in different tissues. Tamoxifen blocks estrogen receptors in breast tissue (therapeutic) while activating them in the endometrium (carcinogenic risk) (Riggs and Hartmann, 2003; Jordan, 2007). Non-selective NSAIDs reduce inflammation at injury sites yet simultaneously damage the gastric mucosa (Ricciotti and FitzGerald, 2011). Beta-blockers slow the heart (desired) but constrict bronchioles (adverse) (Salpeter et al., 2002). Sorafenib inhibits tumor angiogenesis in the liver while causing cardiotoxicity through off-target kinase inhibition in the myocardium (Force et al., 2007).

**FIGURE 1:**
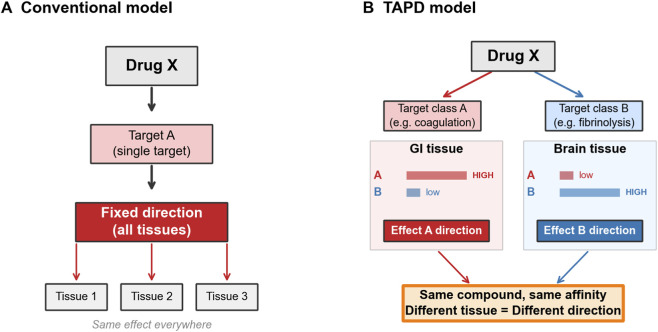
The TAPD concept. **(A)** Conventional pharmacological model: a single-target drug produces the same directional effect across all tissues. **(B)** TAPD model: a multi-target drug engages two functionally opposing target classes (e.g., coagulation vs. fibrinolysis). In tissues where Target class A is highly expressed (e.g., GI tissue), the net effect favors Direction A; in tissues where Target class B dominates (e.g., brain tissue), the same compound produces Direction B. The compound’s intrinsic binding profile has not changed; the tissue determines the pharmacological direction.

Conventionally, each case is treated as a pharmacological curiosity explained by mechanisms unique to the drug in question. We argue that these phenomena are instead manifestations of a single underlying principle. In this Perspective, we propose tissue-autonomous pharmacological direction (TAPD) and introduce a quantitative tool, the Tissue Direction Index (TDI), that predicts when and where a multi-target compound will undergo direction reversal (Figure 1).

## 2 Conventional explanations and their limits

The dominant explanation for tissue-dependent drug effects invokes receptor subtype selectivity. The SERM (Selective Estrogen Receptor Modulator) paradigm is the classic example: tamoxifen’s tissue-selective effects are attributed to differential expression of ER-α and ER-β, combined with tissue-specific coregulator proteins that determine whether a given ER-ligand complex activates or represses transcription (Riggs and Hartmann, 2003; Smith and O’Malley, 2004). This model has proved highly productive, enabling prospective predictions of tissue-selective drug behavior and underpinning the rational design of selective agents across endocrinology and oncology.

However, the receptor subtype model has fundamental limitations when applied to multi-target pharmacology. First, it requires well-characterized receptor subtypes with opposing functional outputs, a condition met by only a small fraction of the druggable proteome (Santos et al., 2017; Overington et al., 2006). Second, the model addresses one target at a time: it explains why tamoxifen acts as agonist *versus* antagonist at ER, but does not address compounds that simultaneously engage dozens of functionally distinct targets, as is common for natural products and designed polypharmacological agents (Hopkins, 2008; Anighoro et al., 2014). Third, although successful within specific receptor families, the subtype model does not generalize across drug classes without bespoke mechanistic characterization for each new target system.

Other explanations include tissue-specific drug metabolism (Zanger and Schwab, 2013), pharmacokinetic concentration gradients (Smith et al., 2010), and epigenetic modulation of target accessibility (Kelly et al., 2010). Each captures a real biological phenomenon, but none generalizes beyond the specific drug or target in question. What remains missing is a computable framework that predicts tissue-dependent pharmacological direction from first principles, applicable across drug classes and tissue types without requiring bespoke mechanistic studies for each new combination.

## 3 Tissue-autonomous pharmacological direction

We propose that when a compound simultaneously engages two functionally opposing classes of targets, its net pharmacological direction in a given tissue is determined primarily by which class is more abundantly expressed locally, rather than by the compound’s intrinsic binding preferences alone.

An analogy is instructive: a driver pressing both the accelerator and the brake simultaneously. Even when the driver applies greater force to the accelerator, a vehicle fitted with a sufficiently larger brake pedal will still decelerate. The driver (compound) has not changed; the vehicle (tissue) determines the direction.

Three conditions are necessary for TAPD to operate:

- **Polypharmacology:** The compound must engage multiple targets simultaneously, a common property of natural products, kinase inhibitors, and CNS-active drugs (Hopkins, 2008; Barabási et al., 2011; Boran and Iyengar, 2010).
- **Functional opposition:** The targets must belong to pathways with opposing biological outputs (e.g., coagulation vs. fibrinolysis, proliferation vs. apoptosis).
- **Tissue-differential expression:** The expression levels of opposing target classes must differ significantly across tissues, a condition abundantly satisfied by transcriptomic atlases such as GTEx (GTEx Consortium, 2020) and the Human Protein Atlas (Uhlén et al., 2015).

When all three conditions are met, the compound’s net effect is no longer a fixed molecular attribute but an emergent consequence of the tissue in which the drug acts.

### 3.1 The tissue direction index

To quantify TAPD, we introduce the Tissue Direction Index (TDI), a dimensionless metric ranging from 0 to 1:

$$\text{Effect}\_A\_{\text{score}}_{\text{tissue}}=\left(1/N_{A}\right)\times \Sigma_{i}\;\left(\left|\Delta G_{i}\right|\times {\text{Expr}}_{i,\text{tissue}}\right)\text{ for targets promoting Effect }A$$

$$\text{Effect}\_B\_{\text{score}}_{\text{tissue}}=\left(1/N_{B}\right)\times \Sigma_{j}\;\left(\left|\Delta G_{j}\right|\times {\text{Expr}}_{j,\text{tissue}}\right)\text{ for targets promoting Effect }B$$

$${\text{TDI}}_{\text{tissue}}=\text{Effect}\_A\_\text{score }/\;\left(\text{Effect}\_A\_\text{score}+\text{Effect}\_B\_\text{score}\right)$$

where |ΔG| represents the absolute binding affinity (from docking, K_i_, or IC_50_ data) and Expr represents the tissue-specific expression level (TPM from GTEx, or protein abundance from HPA). Per-class normalization (dividing by N_A_ and N_B_) prevents count bias when the number of targets differs between functional classes.

- TDI > 0.5: net pharmacological direction favors Effect A
- TDI < 0.5: net pharmacological direction favors Effect B
- Direction reversal: TDI crosses the 0.5 threshold between two tissues

The TDI is deliberately simple, sacrificing mechanistic detail for generalizability: the same formula applies to hemostasis, hormone signaling, kinase networks, or any pharmacological system decomposable into two opposing functional outputs. Several simplifying assumptions merit justification. The use of |ΔG| as a linear proxy is a first-order approximation (binding free energy relates logarithmically to Kd); we retain it because it is widely available and because the sensitivity analysis (Section 4) shows conclusions hold even with uniform |ΔG|. Equal weighting of targets ignores catalytic amplification and network centrality, but pathway-specific weights would require stoichiometric models currently unavailable for most drug-target systems. Per-class normalization (dividing by NA and NB) prevents annotation completeness from confounding biological signal. TDI thus represents a transcriptomic directional proxy rather than a definitive pharmacodynamic predictor. Future extensions may incorporate pathway centrality weighting, z-score normalization, or protein-protein interaction weights (Csermely et al., 2005), but even this minimal formulation captures the core phenomenon.

## 4 Proof of concept: bidirectional hemostatic regulation

The clinical question that motivated this work provided a natural test case (Chen et al., 2026). We identified seven bridging metabolites shared between traditional Chinese botanical drugs classified as both blood-activating and hemostasis-promoting in the Chinese Pharmacopoeia (Chinese Pharmacopoeia Commission, 2020). Six metabolites passed ADMET screening and were docked against 25 hemostatic targets: six coagulation-promoting (F2, F10, F7, F3, PTGS1, PLAT), 12 fibrinolysis-promoting (including the brain-enriched CALM1, GRIN1, and PPP3CA), and seven dual-pathway targets (Chen et al., 2026).

The results revealed a clear dissociation between molecular affinity and tissue-level direction. At the molecular level, all six metabolites exhibited uniform coagulation bias: the binding affinity differential (ΔΔG = mean ΔG_coagulation_ − mean ΔG_fibrinolysis_) ranged from −0.76 to −1.13 kcal/mol (Supplementary Data S1). These values fall within the typical error margin of molecular docking (1–2 kcal/mol) (Li et al., 2019; Wang et al., 2016), and the molecular-level coagulation bias should therefore be interpreted as a trend rather than a definitive finding. Moreover, several of these metabolites, notably quercetin, are recognized pan-assay interference compounds (PAINS) whose apparent binding in computational and *in vitro* assays may partly reflect non-specific interactions rather than genuine target engagement (Baell and Holloway, 2010; Bolz et al., 2021).

At the tissue level, a far larger effect emerged. GTEx expression data showed that coagulation targets were heavily enriched in GI tissues (COL1A1: 175 TPM; COL3A1: 243 TPM; PTGS1: 66 TPM), whereas fibrinolysis targets dominated in the brain (CALM1: 686 TPM; GRIN1: 109 TPM; PPP3CA: 91 TPM) (GTEx Consortium, 2020). When binding affinities were weighted by tissue-specific expression to calculate the Hemostatic Index (HI, a domain-specific instantiation of TDI), the mean HI shifted 4.6-fold, from 0.496 in GI tissue to 0.107 in brain tissue.

A sensitivity analysis confirmed that tissue expression, not binding affinity, drives this shift (Supplementary Figure S1; Supplementary Table S1). When all binding affinities were set to a uniform value, the HI still shifted 4.8-fold between tissues (GI: 0.45; Brain: 0.095), with the pattern entirely expression-driven. Two separable claims emerge from this proof of concept. The primary finding is the expression-driven directional shift: tissue expression differences alone produce a 4.8-fold change in Hemostatic Index, converting a molecular-level coagulation bias into a strongly blood-activating profile in the brain. This claim is independent of docking accuracy and robust to PAINS-related concerns. The secondary observation is that two metabolites, quercetin and eriodictyol, cross the HI = 0.5 threshold in GI tissue when compound-specific affinities are included (GI HI = 0.515 and 0.507 vs. Brain HI = 0.114 and 0.112). This observation depends on docking data and should be regarded as illustrative rather than definitive, given the acknowledged error margins. The 0.5 threshold is a convenient interpretive boundary, not a biologically fundamental value. Alternative target classifications, Monte Carlo propagation of docking error, and an explicit expression-ratio baseline are reported in Supplementary Table S2. In sum, binding affinity fine-tunes the TDI, but tissue expression determines the pharmacological direction.

Cross-disease transcriptomic validation using GEO datasets for ischemic stroke (GSE16561) and ulcerative colitis, a gastrointestinal bleeding–associated mucosal disease (GSE66407) identified four targets (PTEN, HMOX1, RAF1, and MAPK14) that reached statistical significance for differential expression in both conditions simultaneously, with opposing directions between the two diseases (Chen et al., 2026). These targets provide direct evidence that the biological substrate for expression-driven direction reversal exists in clinically relevant disease contexts. More broadly, 38.4% of shared hemostatic targets showed opposite expression directions between the two diseases, a substantial fraction exhibiting the opposing directionality on which TAPD operates.

**FIGURE 2:**
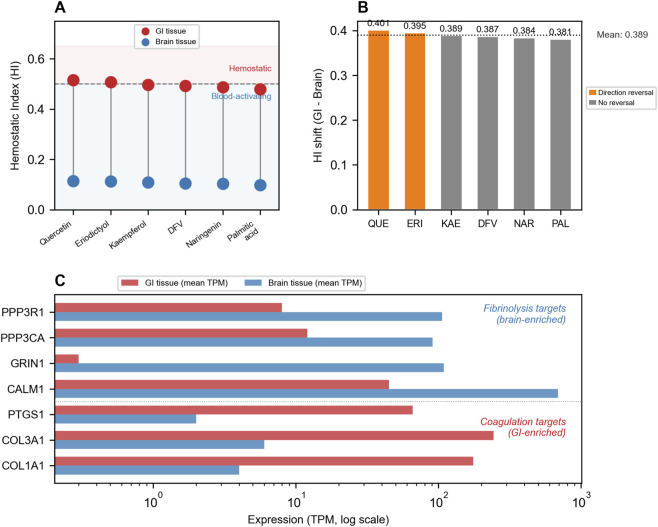
Proof of concept: hemostatic direction reversal. **(A)** Hemostatic Index (HI) for six bridging metabolites in GI tissue (red circles) and brain tissue (blue circles). The dashed line at HI = 0.5 separates the hemostatic (above) and blood-activating (below) zones. Quercetin and eriodictyol achieve true direction reversal (GI HI > 0.5, Brain HI < 0.5). **(B)** HI shift magnitude (GI − Brain) for each metabolite; orange bars indicate metabolites achieving direction reversal. **(C)** Tissue-specific expression (mean TPM, log scale) of key hemostatic targets in GI tissue (red) and brain tissue (blue). GI tissue values represent the mean across five GTEx subregions (colon transverse, colon sigmoid, small intestine terminal ileum, stomach, and esophagus mucosa); direction reversal was consistent across all five subregions. Within-subregion coefficient of variation for individual targets ranged from 0.15 to 0.42. Data from GTEx v8 (GTEx Consortium, 2020).

The proof of concept illustrates a simple but counterintuitive point (Figure 2): tissue expression can override molecular binding preferences to determine the net pharmacological direction.

## 5 TAPD beyond hemostasis

If TAPD is a general principle, it should manifest beyond hemostasis. We examined four well-characterized cases of tissue-dependent pharmacology through the TAPD lens.

The SERM case represents a boundary condition for TAPD. Tamoxifen’s tissue selectivity is governed primarily by ligand-induced receptor conformation and tissue-specific coregulator recruitment (SRC-1 vs. SRC-3), not by differential receptor abundance (Riggs and Hartmann, 2003; Smith and O’Malley, 2004; Shang and Brown, 2002). Because coactivators and corepressors are not direct drug targets, their expression does not map straightforwardly onto the TDI formula, and we do not claim that TDI can quantitatively recapitulate SERM tissue selectivity in its current form. Nevertheless, the SERM paradigm illustrates the broader principle that tissue-specific molecular context determines drug direction, a principle that TAPD formalizes and extends to cases involving direct multi-target engagement. We emphasize that this is a qualitative analogy: the TDI is not computed for tamoxifen, and we present SERM biology as a precedent consistent with TAPD rather than as quantitative evidence for it.

TAPD applies more naturally to cases involving direct multi-target engagement. The GI toxicity of non-selective NSAIDs reflects high constitutive COX-1 expression in gastric mucosa (yielding a TDI biased toward mucosal disruption), whereas induced COX-2 at inflamed joints shifts the TDI toward anti-inflammatory benefit (Ricciotti and FitzGerald, 2011; Vane and Botting, 1998). In tissues with intermediate COX-1/COX-2 ratios, TAPD predicts the net direction from expression data, potentially explaining heterogeneous cardiovascular risk profiles across NSAIDs (FitzGerald, 2003).

The principle extends to newer drug classes. Sorafenib achieves antiangiogenic effects in hepatocellular carcinoma, where VEGFR expression dominates, but its TDI shifts toward cardiotoxicity in the myocardium, where survival kinases predominate (Force et al., 2007; Llovet et al., 2008; Orphanos et al., 2009). Through the TAPD lens, kinase inhibitor cardiotoxicity becomes a predictable consequence of tissue-dependent TDI values computable from cardiac expression atlases (Moslehi, 2016). In immunology, the tumor microenvironment and autoimmune lesions present opposing contexts: compounds targeting shared immunological nodes (JAK/STAT inhibitors, checkpoint modulators) may undergo TAPD-driven direction reversal depending on the local balance of activating *versus* suppressive mediators (Waldman et al., 2020; O’Shea et al., 2015).

## 6 Translational implications

TAPD generates four testable translational hypotheses. First, every multi-target drug has, in principle, a tissue-specific TDI profile across all GTEx tissues; screening approved drugs for unexpected TDI reversal may reveal repurposing opportunities without new chemistry (Pushpakom et al., 2019; Corsello et al., 2017). Second, off-target toxicity can be viewed as unintended TDI reversal, and computing TDI across all tissues during preclinical development could flag tissues at risk of paradoxical effects before they manifest clinically (Blomme and Will, 2016). Third, tumor RNA-seq data combined with binding affinity profiles could yield personalized TDI values, potentially predicting not only whether a drug will be effective but in which direction it will act in a given patient’s tumor (Marquart et al., 2018), extending pharmacogenomics from genotype–drug interactions to expression-profile–drug interactions. Fourth, single-cell RNA-seq atlases (Regev et al., 2017) open the door to TDI computation at cellular resolution, where different cell types within one tissue may show opposing TDI values for the same compound, partly explaining variable drug responses (Mitchell et al., 2021). These applications remain hypothesis-generating; none has been validated experimentally, and predictive benchmarking against clinical outcomes is an essential next step.

To move beyond retrospective reinterpretation, we offer two specific, falsifiable predictions. First, TAPD predicts that sorafenib-induced cardiotoxicity severity should correlate with the patient-specific cardiac expression ratio of survival kinases (RSK2, S6K1) to VEGFR2: patients with higher RSK2/VEGFR2 ratios in myocardial tissue should exhibit greater cardiotoxicity at equivalent drug exposures. This prediction is testable using clinical cohorts with paired tumor and cardiac expression data; cardiac tissue is obtainable from surgical specimens, autopsy, or iPSC-cardiomyocyte transcriptomes. Second, TAPD predicts that among patients receiving the same non-selective NSAID, those with higher gastric mucosal COX-1/COX-2 expression ratios (measurable from routine endoscopy biopsies) should have higher rates of GI bleeding, because a COX-1-dominant expression profile shifts the local TDI toward mucosal disruption. This prediction extends beyond the established drug-class-level observation that COX-1 inhibition causes GI damage (Vane and Botting, 1998; FitzGerald, 2003): TAPD adds a patient-specific, quantitative dimension by predicting that inter-individual variation in mucosal expression ratios, not just drug selectivity, determines GI bleeding risk for the same drug at the same dose. Both predictions involve drugs with well-characterized binding data and no PAINS concerns.

## 7 Limitations

TAPD is a working model, not a universal law, and several boundary conditions deserve explicit discussion. In particular, TAPD models direction within a defined pair of opposing target classes; a tissue-selective effect driven by a genuine off-target outside both classes, such as sunitinib cardiotoxicity via AMPK inhibition, falls outside a given TDI and would require incorporating that target as an additional class.

The most fundamental limitation is that TDI in its current implementation represents a transcriptomic directional tendency rather than a true pharmacodynamic index, because mRNA expression does not equate to functional target availability for several target classes in our proof of concept. For structural extracellular matrix proteins such as COL1A1 and COL3A1, tissue abundance reflects years of protein deposition and remodeling; mRNA levels indicate ongoing synthesis but do not capture the accumulated protein pool that serves as the functional substrate for platelet adhesion (Vogel and Marcotte, 2012). For circulating coagulation factors such as F2 (prothrombin) and F10 (Factor X), which are synthesized predominantly in the liver and reach peripheral tissues via the bloodstream, local tissue mRNA bears little relationship to functionally available protein concentrations at those sites; plasma proteomics or tissue-level activity assays would be more appropriate TDI inputs. For PTGS1 (COX-1), enzymatic activity at a tissue site depends substantially on platelet abundance and activation state, not solely on local transcription. Intracellular signaling proteins such as CALM1 and PPP3CA generally show better mRNA-protein concordance, but post-translational regulation (e.g., calcium-dependent calmodulin activation) introduces additional uncertainty not captured by expression data.

These limitations affect the precision of TDI estimates for specific targets but do not invalidate the underlying principle. The TDI framework is agnostic to data source: replacing mRNA with tissue-level proteomics, phosphoproteomics, activity-based protein profiling, or drug-target residence time data would refine the metric without altering its logic, progressively closing the gap between transcriptomic directional tendency and pharmacodynamic reality. Bulk expression also averages over cell types, so a tissue-level TDI may mask opposing directions among constituent cells; the same logic applies to single-cell expression vectors, the natural next level of resolution. The sensitivity analysis reported above, in which expression alone drove a 4.8-fold HI shift even with uniform binding affinities, confirms that the core phenomenon is robust to substantial perturbation of binding affinity inputs.

A further caveat concerns the proof-of-concept metabolites. Quercetin and eriodictyol are recognized PAINS that can produce false-positive signals in computational and biochemical assays (Baell and Holloway, 2010; Bolz et al., 2021), so their docking-derived affinities should be interpreted accordingly. TAPD itself does not depend on these specific metabolites: the sensitivity analysis showed that the tissue-dependent HI pattern persists under uniform binding, confirming expression as the primary driver. The non-hemostatic examples in Section 5 involve well-validated pharmaceutical agents not subject to PAINS concerns.

TDI also treats targets as independent contributors, yet pathway crosstalk means that engaging target A may alter the functional consequence of engaging target B (Menden et al., 2019). Network-aware extensions incorporating interaction weights, pathway centrality scores, or stoichiometric coefficients from protein–protein interaction data represent natural next steps that could transform TDI from a transcriptomic index into a systems-level pharmacodynamic predictor.

Finally, pharmacokinetics cannot be ignored: a compound predicted to reverse direction in the brain matters only if it crosses the blood–brain barrier (Pardridge, 2005). Of our six metabolites, only DFV and palmitic acid are predicted to be brain-penetrant, so the brain HI describes the direction a compound would exert if delivered, relevant for blood-brain-barrier-permeable analogues or the barrier disruption that accompanies acute stroke. Despite these caveats, the minimal TDI formulation captures a real and underappreciated phenomenon: differences in target expression across tissues can dominate over molecular binding preferences to determine pharmacological direction.

## 8 Concluding remarks

Pharmacology has traditionally treated drug direction as a molecular-level constant. TAPD challenges this view: for multi-target compounds, pharmacological direction depends as much on where a drug acts as on what it binds. While this principle is implicit in network pharmacology and systems biology, TAPD makes three specific contributions: it formalizes expression-driven direction reversal as a quantifiable prediction; it enables cross-tissue comparison as a first-class operation, providing a minimal-parameter index (TDI) that evaluates the same compound across tissues without tissue-specific model calibration; and it shifts the question from pathway engagement to pharmacological direction. We began with a clinical paradox: the impossibility of achieving hemostasis and anticoagulation simultaneously. Compounds with balanced binding profiles undergo automatic direction shifts as they encounter tissues with different target expression landscapes. TAPD remains a hypothesis-generating framework whose predictive value must be established through prospective validation, but the principle that tissue expression determines pharmacological direction may reshape how we approach multi-target pharmacology.

## Data Availability

Publicly available datasets were analyzed in this study. The transcriptomic data are available from the GTEx Portal (GTEx v8; https://gtexportal.org) and the NCBI Gene Expression Omnibus (https://www.ncbi.nlm.nih.gov/geo/) under accession numbers GSE16561 and GSE66407. The processed proof-of-concept data generated for this study are provided in the Supplementary Material (Supplementary Tables S1 and S2 and Supplementary Data S1) and are openly archived at Zenodo (DOI: 10.5281/zenodo.20765877); these data include the molecular docking affinities, target classifications, tissue-specific expression values, and the sensitivity and robustness analyses.
