# Supplementary figures and images for: Tissue-autonomous pharmacological direction: how target expression landscapes convert balanced compounds into tissue-selective agents
Source: Front Pharmacol. 2026 Jun 30;17:1870571. doi: 10.3389/fphar.2026.1870571 (PMC13365337; doi:10.3389/fphar.2026.1870571)

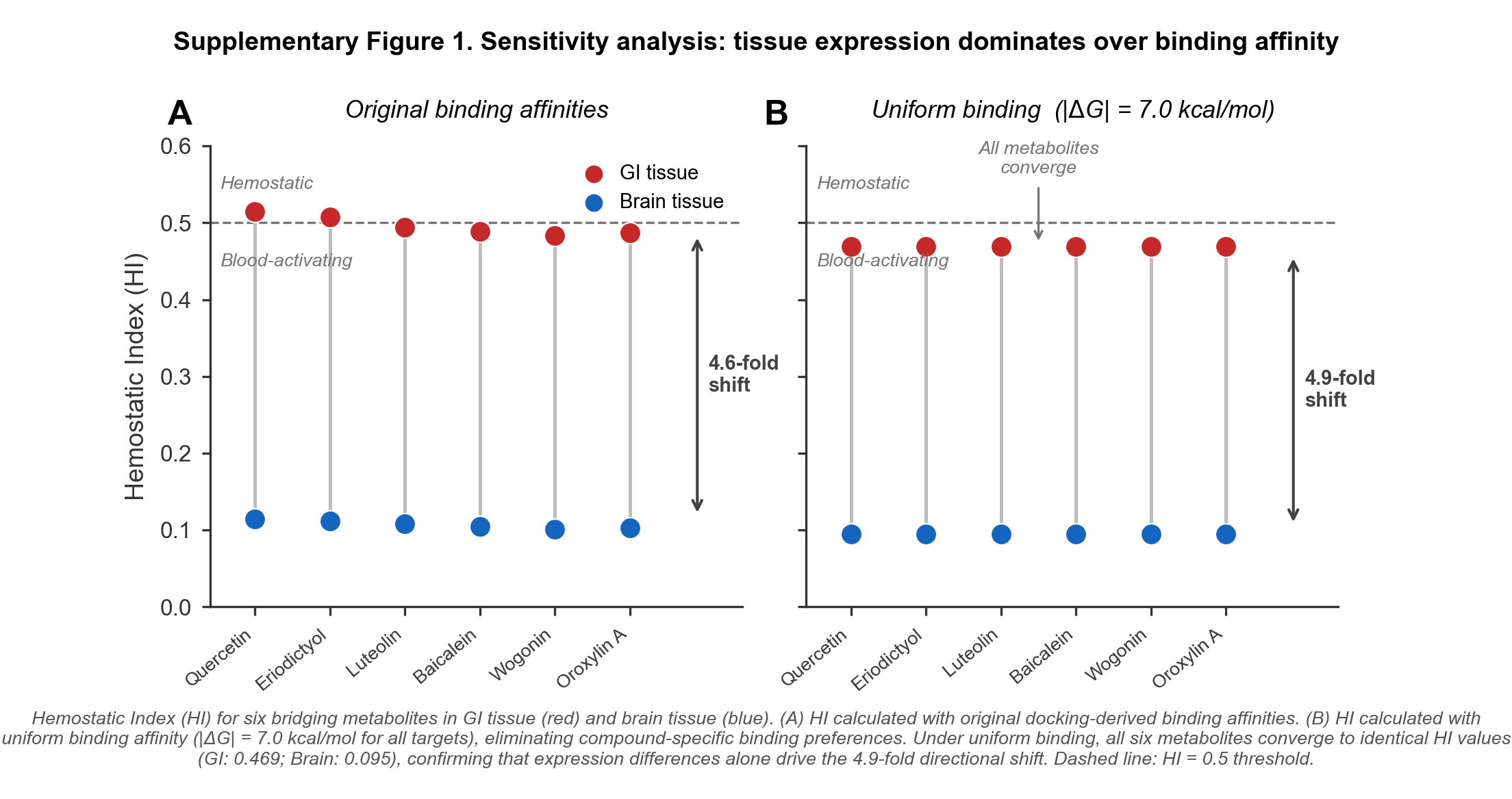

Supplement: Supplementary file 4 [file Image1.PNG]
